# How Do Mechanics Guide Fibroblast Activity? Complex Disruptions during Emphysema Shape Cellular Responses and Limit Research

**DOI:** 10.3390/bioengineering8080110

**Published:** 2021-08-05

**Authors:** Mathew N. Leslie, Joshua Chou, Paul M. Young, Daniela Traini, Peta Bradbury, Hui Xin Ong

**Affiliations:** 1 Respiratory Technology, The Woolcock Institute of Medical Research, Glebe, Sydney, NSW 2037, Australia; Mathew.Leslie@sydney.edu.au (M.N.L.); p.young@mq.edu.au (P.M.Y.); daniela.traini@mq.edu.au (D.T.); 2 Department of Biomedical Sciences, Faculty of Medicine, Healthy and Human Sciences, Macquarie University, Sydney, NSW 2109, Australia; 3 Faculty of Engineering and Information Technology, University of Technology Sydney, Ultimo, Sydney, NSW 2007, Australia; Joshua.Chou@uts.edu.au; 4 Department of Marketing, Macquarie Business School, Macquarie University, Sydney, NSW 2109, Australia; 5 Mechanics and Genetics of Embryonic and Tumoral Development Group, UMR168—Laboratoire Physico-Chimie Curie, Institut Curie, 75248 Paris, France

**Keywords:** emphysema, lung, fibroblast, mechanobiology, 3D lung, inhaled treatment

## Abstract

The emphysema death toll has steadily risen over recent decades, causing the disease to become the third most common cause of death worldwide in 2019. Emphysema is currently incurable and could be due to a genetic condition (Alpha-1 antitrypsin deficiency) or exposure to pollutants/irritants, such as cigarette smoke or poorly ventilated cooking fires. Despite the growing burden of emphysema, the mechanisms behind emphysematous pathogenesis and progression are not fully understood by the scientific literature. A key aspect of emphysematous progression is the destruction of the lung parenchyma extracellular matrix (ECM), causing a drastic shift in the mechanical properties of the lung (known as mechanobiology). The mechanical properties of the lung such as the stiffness of the parenchyma (measured as the elastic modulus) and the stretch forces required for inhalation and exhalation are both reduced in emphysema. Fibroblasts function to maintain the structural and mechanical integrity of the lung parenchyma, yet, in the context of emphysema, these fibroblasts appear incapable of repairing the ECM, allowing emphysema to progress. This relationship between the disturbances in the mechanical cues experienced by an emphysematous lung and fibroblast behaviour is constantly overlooked and consequently understudied, thus warranting further research. Interestingly, the failure of current research models to integrate the altered mechanical environment of an emphysematous lung may be limiting our understanding of emphysematous pathogenesis and progression, potentially disrupting the development of novel treatments. This review will focus on the significance of emphysematous lung mechanobiology to fibroblast activity and current research limitations by examining: (1) the impact of mechanical cues on fibroblast activity and the cell cycle, (2) the potential role of mechanical cues in the diminished activity of emphysematous fibroblasts and, finally, (3) the limitations of current emphysematous lung research models and treatments as a result of the overlooked emphysematous mechanical environment.

## 1. Introduction

### 1.1. Lung Structure Allows for the Cyclical Stretch and Recoil of Inspiration and Expiration

The lung extracellular matrix (ECM) provides the appropriate structure and elasticity required to facilitate respiration and gas exchange via rhythmic inhalation (inflation) and exhalation (deflation) [1,2,3,4]. The highly specialised, 3D organisation of lung ECM scaffolding proteins (namely, collagen and elastin) allows the lung to endure constant cycles of stretch (inflation) and recoil (deflation). This highly specific architectural configuration of scaffolding proteins supports the organisation of multiple lung cell populations and provides the lung with the elasticity and durability needed for cyclic stretch to occur without compromising lung integrity [1,4,5,6,7,8,9]. Additionally, the lung ECM also collectively comprises the lung parenchyma—a region of the lung that supports the airways and regulates gas exchange between the alveoli and blood of the pulmonary capillaries. Importantly, this gas exchange is a direct result of transpulmonary pressures produced by the rhythmic stretch and recoil of inhalation and exhalation [3,10,11], highlighting the vital role the ECM plays in maintaining not only the lung structure but also lung function. Thus, the lung ECM provides the structural support required to maintain cyclical stretch and subsequently exerts stretch-induced mechanical forces (stress and tension) on the variety of cell populations such as fibroblasts that reside within the lung to influence, regulate and determine individual cell functions [6,7,10,11,12,13,14]. This constant stretch, coupled with the inevitable inhalation of foreign particles, places the lung at high risk of insult and injury, and, as a result, the lung ECM requires constant maintenance and repair. Lung fibroblasts are responsible for maintaining the lung ECM by coordinated synthesis and organisation of scaffolding proteins to preserve ECM homeostasis and lung function [15,16,17].

### 1.2. Emphysema Disease Progression Alters Mechanical Cues by Destroying Lung Structure

The progressive destruction of the lung ECM is a core aspect of emphysematous disease progression, as the parenchyma that structurally supports the alveoli air sacs is permanently and abnormally destroyed. The resulting emphysematous lung is therefore characterised by several pathophysiological conditions, including elevated oxidative stress (increased reactive oxygen species (ROS)) [18], a chronic inflammatory environment [19,20], upregulated metallo-matrix proteinases (MMPs) [21] and, most importantly, severe destruction of the lung parenchyma [1,22,23,24,25]. Oxidative stress is known to be caused by inhalation of cigarette smoke, consequently activating the alveolar macrophages through the transcription factor nuclear factor-κB (NF-κB) [18,26]. NF-κB induces the release of pro-inflammatory cytokines such as IL-8 and TNF-α, which, in turn, recruits immune cells to the lung, including CD8+ lymphocytes and neutrophils [26,27]. Persistent inflammatory immune responses and lung injury due to cigarette smoke contribute to maintaining a chronic inflammatory environment within the emphysematous lung [19,20]. Likewise, the release of MMPs by alveolar neutrophils and macrophages becomes upregulated during emphysema, resulting in an imbalance of protease and antiprotease activity that further damages and disorders the emphysematous lung ECM [21]. Emphysematous lung conditions such as these are known to contribute towards disease progression; however, this review will primarily discuss the effects of lung parenchymal destruction.

**Figure 1:**
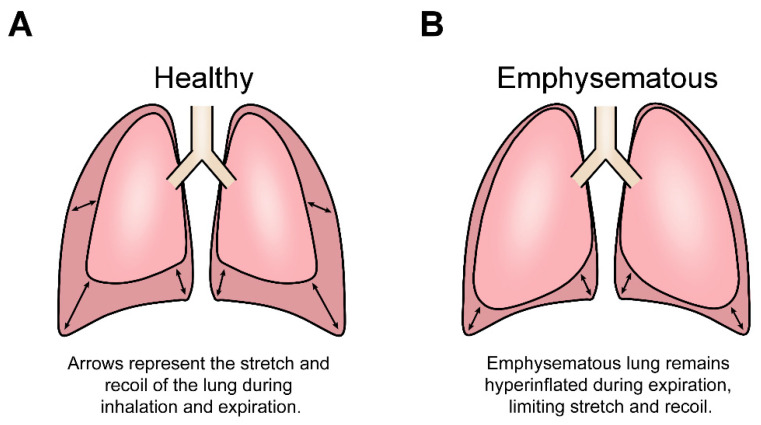
Variations in the mechanical stretch between healthy and emphysematous cells. (**A**) The difference in volume between inhalation and exhalation of a healthy lung. (**B**) The inhalation and exhalation of a lung with emphysematous hyperinflation, a symptom of emphysema. The emphysematous parenchyma loses elasticity, causing the lung to become incapable of fully recoiling between breaths, thus remaining hyperinflated. Lung cell populations experience reduced flux in the stretch during emphysema.

The gradual breakdown of the parenchyma diminishes the ability of the lung to endure the cyclic stretch of breathing, thus resulting in progressively worsening lung function until death [11,23,28]. Consequently, damage to the parenchymal ECM, namely, destruction of ECM proteins collagen and elastin, not only alters the transpulmonary pressure required for gas exchange but perturbs the lung mechanical environment by reducing lung viscoelasticity. In an emphysematous lung, the stretch capacity during inhalation and exhalation is reduced, meaning that patients are unable to inhale the volume of air required, often leading to patients feeling as though they are suffocating [1,20]. Additionally, the reduced viscoelastic properties result in a condition known as ‘lung hyperinflation’, a disease state that occurs when the lung lacks the elasticity to fully recoil during exhalation, trapping air inside the lung (Figure 1). Furthermore, the permanent destruction of the parenchyma during emphysematous progression reduces lung stiffness, measured by the elastic modulus (kPa), therefore permanently decreasing the structural integrity of the lung. While a healthy lung’s stiffness ranges from ~0.5 to 5 kPa, an emphysematous lung’s stiffness ranges from 0.2 to 2 kPa [29]. This shift in lung stiffness is more distinct when the lung is inflated, as lung stiffness varies with lung volume. A simulated model of the lung was used to predict that fully inflated healthy lung tissue achieved a stiffness of 16.8 kPa, whereas fully inflated emphysematous lung tissue reached just 3.8 kPa [30]. Ultimately, these aberrant changes in lung mechanobiology must alter the behaviour and function of the multiple cell populations that reside within the lung, yet little research has examined the consequences of these altered mechanical cues at the cellular level, especially in the context of fibroblasts. Fibroblasts are responsible for repairing and maintaining the structural integrity of the lung ECM, yet the fibroblasts of an emphysematous lung are unable to repair the parenchymal ECM [31]. Thus, the possibility exists that perhaps the altered mechanical environment of the emphysematous lung disrupts the fibroblast ability to respond to and repair the damaged ECM. Interestingly, the insufficient restorative activity of fibroblasts is not yet fully understood and is rarely attributed to the emphysematous mechanical environment, warranting further exploration.

### 1.3. Fibroblasts Maintain Their Mechanical Environment by Maintaining the ECM

**Figure 2:**
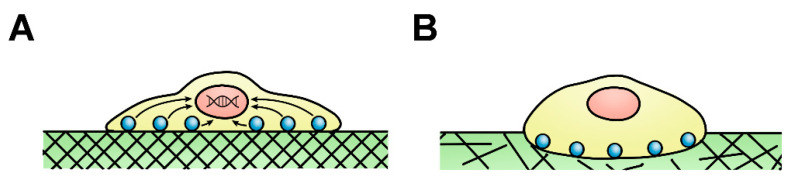
Variations in substrate stiffness between healthy and emphysematous cells. (**A**) Fibroblast seeded on a comparatively stiff healthy lung extracellular matrix (ECM). Substrate stiffness stimulates cell surface proteins to produce a signalling cascade (mechanotransduction) resulting in gene expression. The healthy lung is considered a pliable, low-stiffness organ yet is stiffer in comparison to the emphysematous lung. (**B**) Fibroblast seeded on a soft, emphysematous ECM. ECM scaffolding proteins remain present in the emphysematous lung ECM but are disorganised while cell surface proteins cannot initiate the signalling cascade without mechanical stimulation.

Fibroblasts create an intrinsic positive feedback loop by maintaining ECM homeostasis and, in turn, defining the mechanical nature of the lung parenchyma that then regulates fibroblast activity [16,17,32]. Fibroblasts continuously replace the ECM deteriorated by use and are signalled to enter an activated state by mechanical cues such as heightened stiffness or a pro-inflammatory response in the event of an injury [4,12,33,34]. Fibroblast ECM maintenance is closely tied to the transduction of mechanical cues of resident tissues (Figure 2) as these cues have been shown to regulate many essential fibroblast cellular processes including proliferation, motility and expression of ECM scaffolding proteins. Fibroblasts respond to these mechanical stimuli by converting mechanical stimulation into biochemical signalling cascades that regulate gene expression, and subsequently cell behaviour through a range of signalling pathways such as RhoA/ROCK, COX-2/PGE_2_ and MAPK/ERK [32,33,35,36,37,38,39]. Mechanical environments that experience strong or more frequent stretch forces serve as an activating signal for fibroblast ECM maintenance activity, increasing fibroblast proliferation and mRNA expression of scaffolding proteins used to maintain and repair the ECM as well as reducing apoptosis [35,39,40,41,42]. It has been theorised that fibroblast activity is directed to meet the needs of the local ECM across the body to accommodate a varying mechanical environment [43]. Therefore, upregulation of ECM maintenance activity would theoretically be beneficial to tissues that experience frequent physical stretch, including the lung or vocal folds, as these organs experience more substantial ‘wear and tear’ deterioration, thus requiring greater ECM maintenance to preserve tissue integrity. Somewhat similarly, stiff environments such as cardiac muscle induce a similar upregulation of fibroblast ECM deposition and maintenance to ensure that the resident tissue is maintained at a level of stiffness and durability necessary for that organ [32,39]. Mechanical cues are vital to correct lung function as disrupted ECM conditions and the consequential disrupted mechanical cues dysregulate fibroblast ECM repair recursively. This increasingly dysregulated ECM repair is known to result in pathologies such as idiopathic pulmonary fibrosis (IPF) [44] and maybe a contributing factor towards emphysematous progression [45,46,47]. While there is limited research surrounding the effects of an emphysematous mechanical environment, current research does suggest that fibroblasts detect and respond to mechanical stimuli. Thus, it is conceivable that the aberrant changes to the lung mechanical environment during emphysematous progress may alter fibroblast behaviour and activity.

## 2. Mechanical Cues Guide Fibroblast ECM Maintenance

### 2.1. Mechanical Cues Dynamically Regulate Fibroblast Activity

Fibroblast maintenance of the ECM is closely tied to the detection and interpretation of the mechanical cues of resident tissues [12,45,48,49,50]. Environments that are stiff or experience strong or more frequent stretch forces serve as an activating signal to upregulate fibroblast ECM maintenance activity, proportionally upregulating fibroblast ECM deposition and modelling. This mechanical regulation also drives fibroblast proliferation and increases the gene expression of ECM structural (collagen, elastin, fibrillin, etc.) and non-structural glycoproteins (fibronectin, laminins, etc.) [35,39]. There is a broad consensus that the application of the physical stretch and/or stiffness of tissue can proportionally regulate fibroblast ECM deposition and modelling through upregulating proliferation and the transcription of structural protein genes, including collagen type-1 [6,32,39]. However, the literature surrounding lung fibroblast ECM maintenance is lacking and distinctly hindered by the limited adoption of mechanobiological research techniques.

Mechanical cues exert powerful regulatory effects both within the body and during the in vitro study of mechanosensing cells. Studies have examined the difference in cell cycle progression between fibroblasts seeded within a soft, 3D collagen type-1 matrix and a control group seeded on standard 2D cell culture plastic (2D) [51,52]. The cell cycle of fibroblasts seeded within the collagen matrix was found to be arrested in the early stages of cell division (G_0_/G_1_ phase), with significantly fewer cells actively dividing (S phase) than the cell culture plastic treatment group [51]. Interestingly, the arresting effect was found to be completely reversible when the soft substrate fibroblasts were extracted and seeded on cell culture plastic [52]. The authors of the first study [51] suggested that the discrepancy in the cell cycle may be caused by collagen-dependent suppression of DNA synthesis, yet the notable and obvious changes in the environment (3D vs. 2D), ECM architecture (densely packed collagen network vs. media) or stiffness (~1 kPa [53] vs. 2–4 GPa) [54] were not addressed. Likewise, the authors of the second study [52] did not address the differences in the stiffness and environment, instead suggesting that the difference in the results of the treatment groups is caused by the collagen matrix resembling a tissue-like environment without the need for ECM repair by fibroblasts. However, it is more likely that the mechanism behind the altered cell cycle progression is the discrepancy in the environmental and mechanical conditions of the two substrates, as it has been documented to occur for several additional cell types [55,56,57,58,59,60]. The high stiffness of the cell culture plastic allows cells that were previously arrested in the collagen matrix to quickly alter their behaviour and upregulate the cell cycle and proliferation, displaying cellular activity that may not be biologically relevant. The results of these two studies could be seen as fibroblasts adapting to a different mechanical environment and adjusting their behaviour accordingly. Thus, this result could indicate a substantial difference in the activity of fibroblasts located within the parenchyma and cells that are under typical cell culture conditions. The cause of these differences in cell behaviour can be explained when viewed through the context of mechanobiology, implying that accurate recreation of the local mechanical environment of a cell may be essential to gather biologically and clinically relevant results.

### 2.2. Fibroblast Heterogeneity Demonstrates Mechanical Adaptations

Fibroblasts are deemed to be heterogeneous due to variations in gene expression between populations, described as gene expression signatures, allowing for adaptive responses to a range of mechanical environments [43,61,62]. The ECM composition varies substantially between different organs and is consequently reflected in the differing mechanical cues produced by each tissue environment [12,40,63,64]. Thus, the mechanical variations between tissue environments produce adaptations in fibroblast populations that can provide insights into the mechanobiology of this cell type. This is especially true for organs with a mechanical aspect to their function, particularly stretch-mediated mechanical cues, such as the breathing of the lung, peristalsis of the stomach or the beating of the heart, as these different mechanical environments would have specific ‘local stretch patterns’ as termed by Suki et al. (2013) [47]. These local stretch patterns comprise unique variations in the frequency and amplitude of substrate stiffness or stretch forces between different tissues and often even between different areas of the same organ. These local stretch patterns encourage heterogeneity amongst fibroblasts as the cells adapt to their environment through changes in cell morphology, behaviour and expression profile and are thought to largely be due to epigenetic changes within these fibroblasts [43]. While it has long been known that fibroblasts from different areas of the body are heterogeneous [61], it has only recently been confirmed that even fibroblasts within the same organ can be heterogeneous [65]. This level of fibroblast heterogeneity allows for a highly tissue-specific activity to maintain varying ECM compositions [39,43,62,66]. Organs such as the lung and the vocal folds are exposed to severe and rapidly fluctuating mechanical cues, leading to distinct changes in the gene expression signatures of fibroblasts that could advance the understanding of fibroblast mechanobiology [43].

Heterogenous fibroblast populations employ a wide variety of behavioural and genetic changes in response to mechanical cues to meet diverse ECM maintenance demands across the body. Although rarely examined outside the context of wound healing or disease-associated fibroblasts, limited fibroblast transcriptomic research has directly compared the transcriptome of healthy fibroblasts of varying anatomical origin [43]. Foote et al. (2019) used RNA sequencing to examine the differential expression of fibroblasts derived from seven sites across the body and observed that there were 1271 genes with differential expression when comparing primary human fibroblasts extracted from the lungs and the vocal folds. The strong and rapidly changing stretch forces felt by vocal fold fibroblasts have been shown to increase the expression of FGR and H1P1R, genes associated with cell membrane ruffling. The process of cell membrane ruffling enriches actin at the cell membrane in preparation for cell migration and aids stimuli detection (i.e., mechanical). For vocal fold fibroblasts, the development of actin-rich protrusions allows rapid and discriminate detection of the mechanical and chemotactic cues of the vocal folds, adapting the cells to the increased ECM restorative needs [67,68]. Similarly, this study suggested that the additional mechanical demands of vocal fold fibroblasts have led to the development of further genetic regulatory mechanisms through the upregulated expression of genes such as IGF2 and IGF1R. Although this area of research is still developing, it follows that fibroblasts in high-mechanical stimulation environments across the body adapt to their resultant high ECM maintenance demands by adopting behaviours that increase their capacity for processes such as motility and plasticity. These behavioural or epigenetic adaptations alter the interpretation of mechanical cues in fibroblasts according to the needs of the tissue origin [43,66], signifying the importance of mechanical stimuli and how fibroblast behaviour is dictated by the mechanical environment. These adaptations allow for fibroblasts to migrate and deposit the ECM at an increased rate to maintain ECM homeostasis despite the additional deterioration these tissues may face. Thus, the detection of mechanical cues serves to guide fibroblast behaviour, encouraging niche behavioural adaptations that enable fibroblasts to match the aggressive restorative demands of their local environments. However, this adaptive process relies upon fibroblasts interpreting the typical mechanical cues of a given anatomical origin and does not account for the altered mechanical cues of pathologies such as emphysema.

### 2.3. A Healthy Mechanical Environment Is Essential to Preserve Lung Function and Prevent Lung Pathologies

The regulatory effects of the lung mechanical environment on fibroblasts are indispensable in maintaining lung function; however, the dysregulation caused by altered mechanical cues can encourage pathological ECM maintenance [38,45]. Altered mechanical cues can disrupt the mechanical regulation of fibroblasts, potentially creating a harmful feedback loop as incorrect ECM maintenance may compound disturbances in mechanical cues. An example of this compounding disruption is IPF, a pathological upregulation of injury repair/ECM maintenance in the lung. Excessive ECM deposition results in fibrosis, causing the stiffness of the site of injury to increase, thus causing fibroblast activity to be further upregulated recursively [34,37,39,69,70]. The central role of mechanical regulation in driving IPF progression is well documented and explored [38,45,46,71], yet the mechanobiology of emphysema remains overlooked despite the parallel of a progressively disrupted mechanical environment. The reduced stiffness and stretch of the emphysematous lung have been documented [72] and potentially serve to suppress fibroblast ECM repair, thus resulting in inadequate lung ECM maintenance. The accumulated damage due to insufficient ECM repair would subsequently disrupt the mechanical properties of the lung parenchyma more severely, further disrupting fibroblast ECM maintenance. However, despite evidence of progressive damage to the lung ECM [24,73] and the known link between fibroblast activity and mechanical regulation [7,12,36,74,75], emphysema is rarely examined in the context of mechanobiology.

Interestingly, the changes to fibroblast function that occur in IPF remain reliant on the mechanical environment of the fibroblast long after the onset of the disease. Liu et al. (2015) [37] demonstrated that primary human IPF fibroblasts were able to exit the pro-fibrotic phenotype, displaying cytoplasmic localisation of known mechano-transcription factors YAP and TAZ, thus causing gene expression to align with healthy fibroblasts when seeded on soft (0.4 kPa) 2D substrates without an ECM coating. This suggests that the fibroblast behaviour would return to that of a healthy lung if the mechanical environment could be replaced or repaired during IPF and potentially also emphysema. The reversibility of the IPF fibroblasts, in terms of YAP and TAZ localisation, highlights the importance of correct mechanical environments both for maintaining ECM health and during the research of fibroblasts.

## 3. The Emphysematous Fibroblast Phenotype Could Be Caused by Mechanical Cues

### 3.1. The Effects of Emphysematous Progression on Lung Fibroblasts Correlate with the Effects of Lessened Mechanical Cues

Altered mechanical cues are among the many disease features of emphysema that collectively drive progression, impeding research into the disease through the interplay of these features while destroying the lung [23,47,76]. As mentioned previously, fibroblasts serve as the main producer of the ECM in the body, and thus these cells should theoretically repair and therefore reverse the structural damage of the parenchyma, halting emphysematous progression. However, fibroblasts are unable to heal emphysematous ECM destruction for reasons not fully understood. This failure of emphysematous fibroblasts to repair the damage wrought during disease progression results in the progressive ECM destruction of emphysema and accompanying changes to the mechanical environment [23,31].

Multiple potential explanations for emphysematous fibroblast ineffectiveness have been suggested, each with evidence to support their validity, yet no single theory has been definitively proven to be the sole cause of fibroblast dysregulation [31,47,74,77,78]. One such potential explanation theorises that the volume of ECM damage caused during emphysema may simply outpace the ECM maintenance capacity of lung fibroblasts, requiring fibroblasts to undergo constant proliferation to meet the restorative demand. This increased proliferation is thought to ultimately cause fibroblasts to age prematurely, resulting in senescence of the cells [74]. This theory is supported by the finding that the doubling time of primary emphysematous human lung fibroblasts was nearly 50% longer than that of healthy lung fibroblasts (33.6 ± 2.8 h compared with 24.8 ± 1.4 h, respectively) [74]. This study suggested that the reduced proliferation speed causes fibroblast replacement to fail, potentially due to premature aging of emphysematous fibroblasts. Furthermore, prostaglandin E_2_ (PGE_2_) is a signalling factor known to induce senescence and reduce ECM deposition, contractility and restorative activity in fibroblasts [31]. The level of PGE_2_ is thought to correlate with the severity of airway obstruction during emphysema [77], further increasing the rate of senescence caused by the preterm aging of the cells. The combined effect of PGE_2_-induced senescence and the reduced fibroblast proliferation suggests that fibroblasts are increasingly incapable of meeting the ECM maintenance needs of the lung parenchyma due to a failure of replacement.

The ineffective emphysematous fibroblast activity is also hypothesised to be caused by the chronic inflammatory and hyper-ROS environment of an emphysematous lung as these disease features may disrupt normal fibroblast cell signalling by desensitising fibroblasts to cytokine-mediated chemotaxis [31]. Primary emphysematous fibroblasts were found to perform chemotaxis significantly more slowly than a healthy lung fibroblast control group, displaying a speed of approximately 30% that of healthy fibroblasts [31], therefore severely reducing the ability of emphysematous fibroblasts to locate areas of the damaged ECM. Notably, Noordhoek et al. (2003) [78] observed that the proliferation rate of primary emphysematous human fibroblasts was significantly reduced in the presence of TGF-β1 compared to that of healthy fibroblasts. TGF-β1 was also found to significantly increase the uptake of BrdU (a measure of proliferation) exclusively in healthy fibroblasts, yet it induced no significant effect on the BrdU uptake of emphysematous fibroblasts. The inability of TGF-β1 to stimulate proliferation suggests that emphysematous fibroblasts may be less or no longer responsive to inflammatory mediators, potentially due to a desensitisation of emphysematous fibroblasts caused by their chronic inflammatory environment. However, a less prevalent theory is that the disruption in fibroblast signalling is due to the altered mechanical environment of an emphysematous lung. Given the myriad of changes to the lung environment during emphysematous progression, it is more than likely a combination of all these hypotheses that causes emphysematous fibroblast dysregulation. However, given the recent understanding of the importance of mechanical properties in dictating cell behaviour and activity, the altered mechanobiology of the emphysematous lung warrants not only acknowledgement but also further research as a possible cause of fibroblast dysregulation.

### 3.2. The Complexity of the Emphysematous Environment Limits Research into the Disease

The many features of the emphysematous lung create a complex interplay of pathological influences on fibroblast function and highlight the difficulty of studying emphysema as the precise cause(s) of the aberrant changes to cell function is (are) challenging to differentiate given the many possibilities. Aspects of emphysema such as the chronic inflammatory environment, the presence of ROS and others are known to interfere with fibroblast ECM maintenance [79] and have been studied heavily, yet the cause of fibroblast inactivity remains unknown. Interestingly, the altered mechanical environment of an emphysematous lung remains underexplored and is rarely accounted for when studying emphysematous fibroblasts or progression. The exact effects of the emphysematous mechanical environment on fibroblasts represent a distinct gap in the scientific literature surrounding emphysematous disease progression, having been explored far less than other aspects of the disease. Therefore, it is impossible to draw definite conclusions on the importance of mechanical cues on fibroblast function in emphysema as very few studies examine the disease in a mechanobiological context [28,47,71]. Similarly, few in vitro studies demonstrate the differences in fibroblast behaviour between emphysematous and healthy fibroblasts, largely examining the changes only in senescence, proliferation and cell motility in response to cytokines or ROS. Despite the limits of the currently available research, notable parallels can be made by comparing studies that examine in vitro primary emphysematous fibroblasts and others that explore the effects of mechanical cues on healthy fibroblasts. These studies demonstrate a correlation between the effects of reduced mechanical cues and emphysema, causing a reduction in proliferation and an increase in senescence, suggesting that reduced mechanical cues may be a contributing factor towards inactive emphysematous fibroblasts. Fibroblast mechanobiology in the context of emphysema is currently a gap in the scientific literature surrounding the disease but may yet offer new avenues of research and potential treatments.

## 4. The Mechanical Nature of Emphysema Limits the Study and Treatment of the Disease

### 4.1. In Vitro Models of Emphysema Rarely Replicate the Lung Mechanical Environment

Emphysema is a challenging disease to study due to the limitations in current disease models to accurately replicate the complex interplay of multiple cell types, parenchymal integrity, inflammation and breathing mechanics that all contribute towards emphysematous progression. This is best encapsulated in the frequent use and publication of in vitro emphysema models that rely on seeding a single cell type, whether it be bronchial epithelia, alveoli or fibroblasts, directly on standard cell culture plastic or glass without first coating with physiologically relevant ECMs to replicate a biologically relevant elastic modulus [51,78,80,81,82]. The disparity in stiffness between standard cell culture methods (2–4 GPa) [54] and the emphysematous lung (0.2–2 kPa [29]) results in disease models that are several orders of magnitude stiffer than the mechanical environment of the lung. Likewise, many of these in vitro models lack ECM proteins, are 2D and do not replicate the cytokine signalling between cell populations of the in vivo emphysematous environment, resulting in the loss of both environmental and mechanically mediated signalling [40]. Importantly, these discrepancies between in vitro emphysema models and the in vivo emphysematous environment may confound emphysematous research. These discrepancies are a potential source of error as the differences in fibroblast activity due to cellular signalling or mechanical cues are not replicated in vitro, highlighting the possibility that data generated from in vitro studies that do not consider the mechanical environment may not be physiologically relevant. Furthermore, as most treatment approaches for lung diseases, including emphysema, revolve around the identification of single-target molecule therapeutic development, developing therapies based on possibly ill-identified signalling pathways may result in flawed treatment approaches. Thus, to properly understand the molecular mechanisms that underpin emphysematous progression, we must first understand and fully replicate the in vivo molecular environments in vitro.

The compounding, progressive and destructive nature of emphysema transforms the environmental and mechanical regulation of lung cell populations, disrupting the maintenance of lung parenchymal homeostasis. The destruction of the ECM and reduced cyclical stretch culminate in a severely reduced breathing capacity, a key feature of disease progression [83]. As mentioned previously, substrate stiffness has been shown to regulate cellular processes such as differentiation, proliferation and structural protein deposition in fibroblasts, resulting in the biological relevance of these emphysematous models being called into question. Similarly, stretch and strain forces are often not included, nor considered, in emphysematous models, eliminating yet another key mechanical cue that may govern fibroblast cell activity. It is important to note that in vitro lung models can easily mimic some, if not all, of the emphysematous mechanical environment; substrate stiffness can be replicated using hydrogels of defined stiffnesses [84], while some parenchymal properties can be replicated by simply coating substrates with ECM proteins such as Matrigel, collagen or elastin [85,86]. However, these models are rarely designed to also integrate stretch/strain forces. To incorporate stretch and strain forces, cells must be seeded on a 2D elastic membrane such as polydimethylsiloxane [87] that can then be cyclically stretched to simulate mechanical stresses such as cyclic stretch during breathing. Importantly, these mechanical stretch devices can be purchased commercially or made in-house: Flexcell-type devices [88], lung-on-chip [89] and ‘cell stretchers’ [90,91,92,93]. Additionally, stretch and strain are incorporated into 3D models of the lung through more complex mechanisms that frequently apply biaxial strain by incorporating clamps or embedded anchors within a 3D cell culture matrix. However, these 3D models of the lung are less frequently used and often made in-house [94,95,96]. Although in vitro emphysema models require substantial effort and resources to accurately model the disease, recapitulating the mechanical environment in experimental design and model development better ensures the physiological relevance and validity of results. This is particularly true during the development of novel therapies for diseases such as emphysema, as without mimicking the appropriate in vivo environment in vitro, the results are likely to be confounded, impeding research progress and potentially explaining why novel therapies for emphysema are often ineffective when tested in preclinical trials.

### 4.2. Altered Emphysematous Lung Mechanobiology Is Inadequately Represented in In Vivo Models of Emphysema and Is Not Reversed by Current Treatments

Unlike in vitro models, in vivo models of emphysema can more closely replicate the mechanical environment of the lung; however, they are subjected to different limitations, further complicating the process of developing treatments for emphysema. In vivo models of the disease have been developed from a wide range of animals, with the most common being murine models of emphysema [97]. These models commonly achieve the emphysematous disease state through genetic modification [98], exposure to cigarette smoke [99] or by applying proteases directly to the lungs [100]. However, murine models are unable to simulate late-stage emphysema (defined as GOLD (Global Initiative for Chronic Obstructive Lung Disease) stage 3 or 4 emphysema) as mice fully recover or perish before the disease can mimic what is considered late-stage emphysema in humans [101]. This ability of mice to recover from emphysema calls into question the validity of using murine models of emphysema, as the capacity of mice to reverse the compounding cycle of ECM destruction raises several additional questions. Perhaps mice do not experience a form of emphysema that can be extrapolated to humans. Perhaps mouse fibroblasts are not mechanosensitive or are mechanically regulated via alternate or compensatory pathways to humans. The irreversible nature of emphysematous damage is a core concept of the disease [81,102], meaning the inability of murine models to recreate this aspect of emphysema is a notable limitation. This limitation not only impedes the understanding of human emphysema disease progression but is also a severe limitation in identifying single-molecule targets for novel therapies. There is a consensus in the literature that, unlike humans, mice undergo lung regeneration [81,103,104], theorised to be achieved through the stimulation of basal or alveolar type 2 cell proliferation due to biochemical factors [81,105,106], although the mechanism that allows this regeneration to occur has yet to be definitively determined. Mouse lung regeneration is rarely studied in the context of in vivo mouse models of emphysema, though a mechanobiology-based comparison of healthy and regenerating emphysematous murine fibroblasts may reveal new pathways of lung repair and assist in the discovery of new pharmaceutical targets to treat emphysema.

Lung mechanobiology and how it regulates fibroblast activity are currently underrepresented or overlooked in many normal or diseased in vitro lung models, thus highlighting this issue as one of the many limitations that contribute to a lack of treatment success. Pharmaceutical treatments of emphysema have primarily focused on managing symptoms rather than the cause of the disease, namely, therapies aimed at reducing bronchial constriction and inflammation to maintain lung function [107]; however, this does not halt disease progression, nor does it treat the underlying cause(s). Thus, surgical interventions are often the best option for emphysematous patients, although these too are unable to cure emphysema, except for those patients undergoing single or bilateral lung transplants [108]. Lung volume reduction surgery (LVRS) and bronchoscopic lung volume reduction both alleviate lung hyperinflation and offer superior outcomes to pharmaceutical treatment. However, neither treatment can address the altered mechanical environment of the emphysematous lung by halting the compounding cycle of ECM destruction in emphysematous progression [109,110]. LVRS requires the resection of hyperexpanded highly diseased areas of lung tissue, allowing the remaining healthier lung tissue to fill this space during inhalation and thereby improving lung function for the remaining lung tissue [111]. Similarly, bronchoscopic lung volume reduction achieves the same effect through one-way valves that block the passage of air to highly diseased sections of the emphysematous lung [112]. Currently available surgical and pharmaceutical treatments alone are inadequate to cure emphysema and are limited by their inability to fully restore the lung mechanical environment. Although mechanobiology-based therapies are not a novel concept, current mechanobiological treatments such as stress shielding devices are still emerging yet are frequently inapplicable to mechanical dysregulation inside of the body [113]. Alternative mechanobiology-based treatment could potentially involve chemically disrupting mechanotransduction signalling pathways, a process rarely explored due to the risks of disrupting essential cellular functions, such as tissue development. However, it may be possible for emphysematous fibroblasts to regain their ECM maintenance activity in response to the mechanical cues of a healthy lung, supporting a possibility of a short-term treatment that allows the body to heal the emphysematous lung [52]. However, the development of a treatment capable of restoring fibroblast activity, such as a single-target molecule drug, can only be predicated upon accurate modelling of both the disease emphysema and the emphysematous mechanical cues. The study of emphysema in the context of mechanobiology may offer new avenues of research and possibly curative treatments through advancing the understanding of complex biochemical models of disease.

## 5. Conclusions

The scientific literature agrees that fibroblast function is governed by mechanical cues, resulting in pathologies when these cues are disrupted [45,46]. However, the relationship between fibroblast function and the mechanical environment of the emphysematous lung remains under investigation, despite the well-established mechanical nature of emphysema. A hallmark of emphysematous progression is the inability of fibroblasts to maintain ECM homeostasis, resulting in a compounding cycle of destruction that leads to an increasingly altered lung mechanical environment. Comparable mechanical cues to the emphysematous lung, such as reduced stretch and stiffness, have been observed to reduce fibroblast differentiation, proliferation and gene expression of structural proteins [32,35,36,37,38]. Notably, the changes shown in primary human emphysematous fibroblasts demonstrate changes in cellular behaviour that correlate with the changes to fibroblasts experiencing reduced mechanical cues, such as decreased substrate stiffness and stretch forces [31,32,74,114]. Although these changes are likely influenced by the other aspects of the emphysematous lung environment, this correlation indicates that altered mechanical cues are likely a key driving force for the changes that occur within emphysematous fibroblasts. Models of the lung that replicate the mechanical environment of an emphysematous lung will be essential to bridge the gap in the scientific literature and enhance the current understanding of the disease, potentially revealing avenues of research that not only treat symptoms but may reverse and cure emphysema.

## Data Availability

Not applicable.
